# Androgen Receptor Regulates the Growth of Neuroblastoma Cells *in vitro* and *in vivo*

**DOI:** 10.3389/fnins.2017.00116

**Published:** 2017-03-07

**Authors:** Junyan Sun, Dongmei Wang, Lianying Guo, Shengyun Fang, Yang Wang, Rong Xing

**Affiliations:** ^1^Department of Pathophysiology, College of Basic Medical Sciences, Dalian Medical UniversityDalian, China; ^2^Department of Experimental Functionality, College of Basic Medical SciencesDalian, China; ^3^College of Integrative Medicine, Dalian Medical UniversityDalian, China; ^4^Center for Biomedical Engineering and Technology, Department of Physiology, Department of Biochemistry and Molecular Biology, University of Maryland, School of MedicineBaltimore, MD, USA

**Keywords:** neuroblastoma, androgen receptor, cell proliferation, MDV3100, ARN-509

## Abstract

**Background:** Neuroblastoma is the most common extracranial tumors in children. At present about the true etiology of neuroblastoma is unclear and many studies have tried to find effective treatments for these primary malignant tumors. Although it has been illustrated that androgen receptor (AR) was expressed in neuroblastoma cells in some former reports, the biological role of androgen receptor in the development of neuroblastoma is not fully understood.

**Methods:** Androgen (R1881) and the antagonists of androgen receptor (MDV3100 and ARN509) were used to study the role of the androgen receptor signaling pathway *in vitro* and *in vivo* on SH-SY5Y and Neuro-2a (N2a) cell lines.

**Results:** We found that AR expression showed an R1881 dose-dependent manner in neuroblastoma cells *in vitro* and R1881was able to increase, while both antagonists of androgen receptor (MDV3100 and ARN509) significantly decrease, the proliferation, migration, invasion and sphere formation of SH-SY5Y and N2a cells. Moreover, androgen promoted the growth of N2a tumor *in vivo*. However, when androgen receptor (AR) was effectively knocked down in the two cell lines by siRNA, either promoting or inhibiting effect of the androgen or androgen receptor antagonists, respectively, was attenuated.

**Conclusion:** Our results suggested that androgen receptor may involve in the progression of neuroblastoma as well as provided insight into a new target for the diagnosis and treatment of neuroblastoma patients.

## Introduction

Neuroblastoma (NB) is the most common extracranial solid tumor in children, accounting for about 8–10% of childhood cancers and for about 15% of cancer deaths in children (Ho et al., 2016). The incidence of neuroblastoma is highest in the first year of life and the incidence rates declines considerably thereafter and cases are rare beyond the 10th birthday. It shows aggressive local growth and metastasizes to regional lymph nodes, liver, bone marrow, and bone cortex (Roy Choudhury et al., 2012). Tumors in infants <1 year of age often regress spontaneously and usually have an excellent prognosis, whereas those in older patients are aggressive, leading to a fatal outcome (Su et al., 2015). Despite major advances in therapies, neuroblastoma is still associated with a high morbidity and mortality. Thus, novel diagnostic, prognostic, and therapeutic approaches are required, mainly to improve treatment outcomes of high-risk neuroblastoma patients (Vella et al., 2016).

The cause of neuroblastoma is still unclear, although previous researches mainly focused on the hedgehog pathway, NF-κB pathways (Chaturvedi et al., 2016), mTOR pathway (Mei et al., 2013) and PLK1 pathways (Mao et al., 2009). However, the role of androgen receptor (AR) signaling pathway in molecular pathogenesis of neuroblastoma is rarely reported.

The early studies have shown that SH-SY5Y cells expressed AR by Northern blot analysis using a 32P-labeled probe derived from a human AR cDNA (Yerramilli-Rao et al., 1995). Moreover, it has been reported that androgen can induce tubulin isoforms' upregulation in neuroblastoma cells (Butler et al., 2001). So we speculate whether AR can regulate the growth of neuroblastoma as it functions in prostate cancer cells.

The androgen receptor (AR) is a nuclear receptor that exerts its effects on cells through both classical genomic mechanisms and rapid non-genomic actions (Ciupek et al., 2015). Androgen is mainly secreted and synthesized by testicular, adrenal glands, and ovaries can secrete a small amount. While the most common primary site of neuroblastoma is the retroperitoneum (adrenal gland more often than paraspinal ganglia; Kushner, 2004). The adrenal androgens (AAs), normally secreted by the fetal adrenal zone, the zona fasciculata, and the zona reticularis of the adrenal cortex are steroid hormones with weak androgenic activity. AAs do not appear to play a major role in the fully androgenized adult man, whereas they seem to play a role in the adult woman and in both sexes before puberty (Sidiropoulou et al., 2000). In addition, the incidence was slightly higher in males than that in females (Spix et al., 2006; Navalkele et al., 2011).

Based on the situation that the expression and activity of AR has not been well characterized in neuroblastoma tumor cells and tumors, in our study, we investigated the effect of R1881 (a synthetic AR agonist; Patel et al., 2015) and two AR antagonists, MDV3100 and ARN-509, on cell viability, invasion, mobility and sphere-formation in the neuroblastoma cell lines SH-SY5Y (human neuroblastoma cell line) and Neuro-2a (mouse brain neuroblastoma, N2a), respectively. MDV3100 and ARN509 are two AR antagonists for the treatment of prostate cancer patients. MDV3100 (Enzalutamide, brand name Xtandi), it is a novel oral anti-androgen (Chandrasekar et al., 2015) approved by FDA in 2012 (Schweizer and Yu, 2015). ARN509 is another second generation of androgen receptor antagonists and mainly used in the treatment of castration resistant prostate cancer (Wang et al., 2016). The preliminary clinical results confirmed its security.

## Materials and methods

### Cell culture and treatments

SH-SY5Y, N2a, HeLa, and RAW264.7 were cultured and maintained in Dubelcco's Modified Eagle Media (DMEM) (Gibcol) supplemented with 10% fetal bovine serum (FBS) and 1% Penicillin/Streptomycin (Hyclone). For treatment with vehicle (DMSO), R1881 or ARN-509 and MDV3100, both obtained from APExBio, TX, USA, cells were grown in phenol red-free DMEM (Gibco) medium supplemented with 10% (or indicated concentration, v/v) dextran-coated charcoal stripped FBS (Biological Industries, Israel) for 72 h prior to treatment. Cells were incubated at 37°C in a humidified incubator containing 5% CO_2_. To inhibit AR expression, siRNAs targeting full length AR (siARa, 3′- AAGACGCUUCUACCAGCUCAC -5′; siARb, 3′- AAGAAGGCCAGUUGUAUGGAC -5′; siARc, 3′-GACCUACCGAGGAGCUUU-5′) or siRNA control ordered from GenePharma (Shanghai, China) were transfected using Lipo2000 transfection reagent (Invitrogen) in accordance with the manufacture's protocol.

### Western blots

Cell lysis, protein extraction, and immunoblotting were performed as described previously (Wang et al., 2011).

### MTT/cell viability assay

The proliferative effects of R1881 and the anti-proliferative effects of MDV3100 and ARN509 were measured *in vitro* by using MTT assay. Briefly, the cells were seeded into 96-well plates at a density of 4,000 cells/well. After treatment, the medium was replaced with fresh culture medium contained 0.5 mg/ml MTT reagent and incubated at 37°C for 4 h. Subsequently, the supernatant was aspirated, and cells were lysed in 200 μl DMSO for 10 min at 37°C. The optical density (OD) was measured at 490 and 570 nm using a plate reader. Each experiment was performed in triplicate.

### Immunofluorescence

SH-SY5Y and N2a cells were fixed with 4% paraformaldehyde in 1X PBS at 4°C for 30 min. The slides were incubated with rabbit anti-AR antibody (Santa Cruz) for 1 h, followed by 1 h incubation of TRITC anti-rabbit IgG antibody (Thermo) and stained with DAPI (for the nucleus). Samples were mounted using anti-fade mounting medium, and then visualized with a fluorescent light microscope.

### Wound healing assay

SH-SY5Y and N2a cells were cultured in six-well plates (5 × 10^5^cells/well) and incubated until they reached 90–100% confluence. SH-SY5Y and N2a cells were then maintained in phenol red-free DMEM with 2.5% cFBS (charcoal stripped FBS) or 5% cFBS, respectively, in order to minimize the cell proliferation. A sterile 20 μl tip was used to create scratch wounds of the same width on each monolayer. The plates were then washed twice with phosphate-buffered saline (PBS) to remove the detached cells. Photos were taken at 0, 24, and 48 h, and the distance traveled by the cells enumerated the closure of the wounds. Each experiment was performed in triplicate.

### Trans-well invasion assay

The cells were seeded in the top chamber of Matrigel™-coated inserts (pore size: 8 μm; Falcon) placed in 24-well plates (2 × 10^4^cells/well for SH-SY5Y), while a medium supplemented with 10% cFBS (charcoal stripped FBS) was used as a chemo-attractant in the lower chamber. The wells were coated with 100 μl of Matrigel™ (BD Bioscience) at a dilution of 1:40 (Matrigel: serum-free medium) and air-dried overnight in a biosafety cabinet. The cells were allowed to invade through the Matrigel™ for 48 h at 37°C in a 5% CO_2_ incubator. Cells that did not invade were scraped off with a cotton-tip applicator while the invading cells were fixed and stained with 0.005% crystal violet. The number of invading cells was counted under a light microscope (x10 objective) from three fields for each well. Each experiment was performed in triplicate.

### 3D culture and sphere-formation assay

Single N2a cell suspension was suspended in Matrigel™/serum free DMEM (1:1) at a concentration of 2 × 10^3^cells/well or 4 × 10^3^ in a total volume of 50 μl. The solution was then plated gently individual wells of a 96-well plate and allowed to solidify for 1 h at 37°C. One-hundred microliter of phenol red-free DMEM with 5% cFBS was added gently to the each well and the media (containing the treatment) was changed every 2–3 days. The numbers and morphology of colonies were then counted and observe under a microscope. Each experiment was performed in triplicate.

### Soft agar colony forming experiment

Warm (37°C, 500 μL per well) base agar solution (1.5% agar) in 1 × DMEM complete culture media) was poured into each well of 24-well plate. The base layer was allowed to solidify at 4°C for 30 min. Then 500 μL of a warm (37°C) top agar solution consisting of 8,000 SH-SY5Ycells in 0.7% agar + 1 × DMEM complete tissue culture media was added over the base layer. The 24-well plates were then incubated at 37°C in the humidified incubator for 3 weeks; 500 μL of fresh media was added every 3 days without disturbing the cells. After 3 weeks, the colonies were stained with 0.005% crystal violet. The numbers and morphology of colonies were then counted and observe under a microscope. Each experiment was performed in triplicate.

### Animal xenograft models

To evaluate the efficacy of androgen promoting neuroblastoma cells growing *in vivo*, 10^6^ N2a cells in 10 μl of PBS were injected into the exposed adrenal glands of immunocompromised mice. Five days prior to N2a cells inoculation, 10 out of 15 of the male mice were castrated to eliminate the androgen impact of the testes. Meanwhile 5 intact male mice were of sham surgery. Fifteen male and 12 intact female 4-week-old mice were anesthetized, the left flanks were prepared in sterile fashion, and transverse incisions were performed to expose the left kidneys and the adrenal glands. The mice were then intraperitoneally injected with testosterone propionate (TP, 0.5 mg/kg body weight) dissolved in sesame oil (vehicle) or merely the vehicle. Thus, all the mice were divided into 5 groups: castrated male TP group (*n* = 5), intact male vehicle group (*n* = 5), castrated male vehicle group (*n* = 5), intact female TP group (*n* = 6) and intact female vehicle group (*n* = 6). Each group was treated every 3 days for 2 weeks and then sacrificed. To evaluate the effect of androgen on the survival time of N2a bearing mice, 13 castrated male mice were injected with 2 × 10^6^ N2a cells into the adrenal glands. One week after the inoculation, the mice were randomly separated into two groups and then injected every 3 days intraperitoneally with testosterone propionate (*n* = 6) or the vehicle (*n* = 7), respectively. The cumulative survival curve was generated by SPSS 17.0.

### Ethics approval

All mice experiments were carried out with ethical committee approval and met the standards required by the Dalian Medical University Animal Care and Use Committee guidelines.

### Data analysis

Statistical analysis was performed using GraphPad Prism version 5. The significance of the data was analyzed using a Student's *t*-test, and *p*-values of *p* < 0.05 (^*^), *p* < 0.01 (^**^) and *p* < 0.001 (^***^) were considered significant and highly significant, respectively.

## Results

### AR expression in SH-SY5Y and neuro-2a cells was R1881-dose-dependent

**Figure 1:**
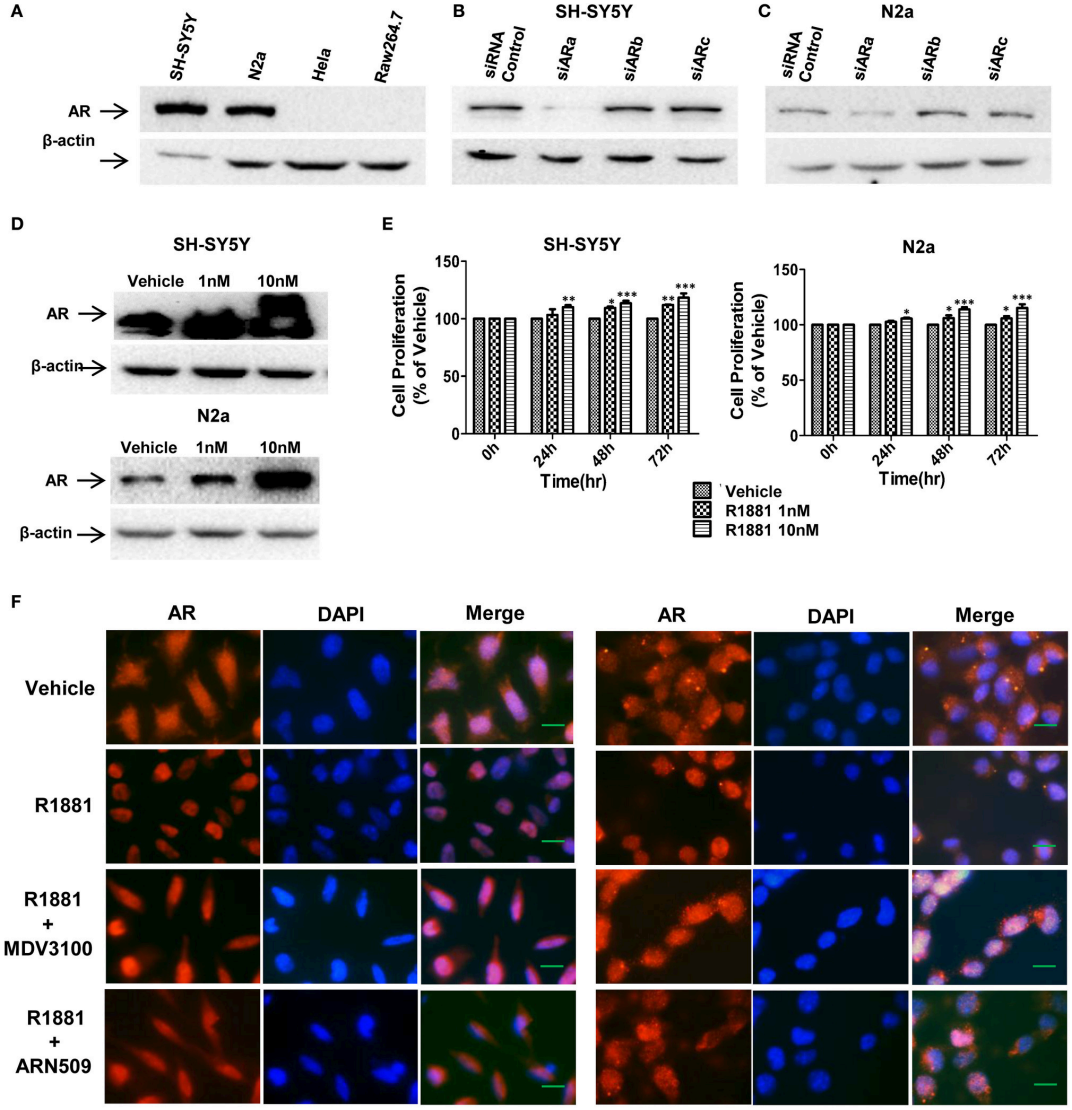
**AR expression in SH-SY5Y and Neuro-2a cells was in a R1881 dose-dependent manner. (A)** Neuroblastoma cells (SH-SY5Y and N2a), human cervical cancer cell line HeLa and mouse leukaemic monocyte macrophage cell line Raw264.7 were lysed and AR was determined by western blot assay (30 μg total protein per lane). All the cell lines werecultured in DMEM supplied with 10% FBS. **(B,C)** Neuroblastoma tumor cells (SH-SY5Y and N2a) were transfected with AR siRNAs (siARa, siARb, siARc), after 72 h cells were lysed and AR was determined by western blot assay. Actin served as internal control. **(D,E)** AR expression and cell viability induced by R1881 were in a dose-dependent manner. **(D)** Cells were treated with DMSO or 1 nM or 10 nM R1881 for 72 h. Actin served as internal control. **(E)** R1881-dose-dependent cell viability was determined using MTT assay. Results are expressed as a percentage of the treated group compared to its control. Data represent an average of three independent experiments. The data are reported as mean ± SD (^*^P < 0.05, ^**^P < 0.01, ^***^P < 0.001). (**F**) R1881 enhanced and AR antagonists impaired AR nuclear-localization. Representative fluorescent microscopic images of SH-SY5Y and N2a cultured (cFBS) and treated with DMSO (vehicle), R1881 (10 nM), MDV3100 (10 μM) combined with R1881 (10 nM), or ARN509 (10 μM) combined with R1881 (10 nM) for 1 h. Scale bar = 10 μm.

To determine the expression level of AR protein in neuroblastoma and other cell lines, neuroblastoma cell lines (SH-SY5Y and N2a), human cervical cancer cell line HeLa and mouse leukaemic monocyte macrophage cell line Raw264.7 were analyzed with Western blotting assay. Neuroblastoma cell lines exhibited high level of AR expression while no AR expression was detected in HeLa or Raw264.7 cells (Figure 1A). Western blotting result also showed that siRNA(a) targeting full length AR significantly reduced the AR expression in SH-SY5Y and N2a cells (the siARa target sequences are similar in both human and mouse, Figures 1B,C). Thus, siARa was applied for knockdown of AR in the following experiments.

Androgen-induced AR expression was also analyzed. AR expression and proliferative effect of R1881, a synthetic androgen as the AR agonist, in both cell lines were in a dose-dependent manner (Figures 1D,E). Furthermore, translocation of AR from cytoplasm to nucleus upon ligand-binding can be reduced by AR antagonist (Figure 1F). Intriguingly, with vehicle treatment, AR expressed in both the cytoplasm and nucleus suggesting the constitutive AR activity in neuroblastoma cells.

### R1881 promotes and AR antagonists inhibit the cell proliferation of neuroblastoma *in vitro*

**Figure 2:**
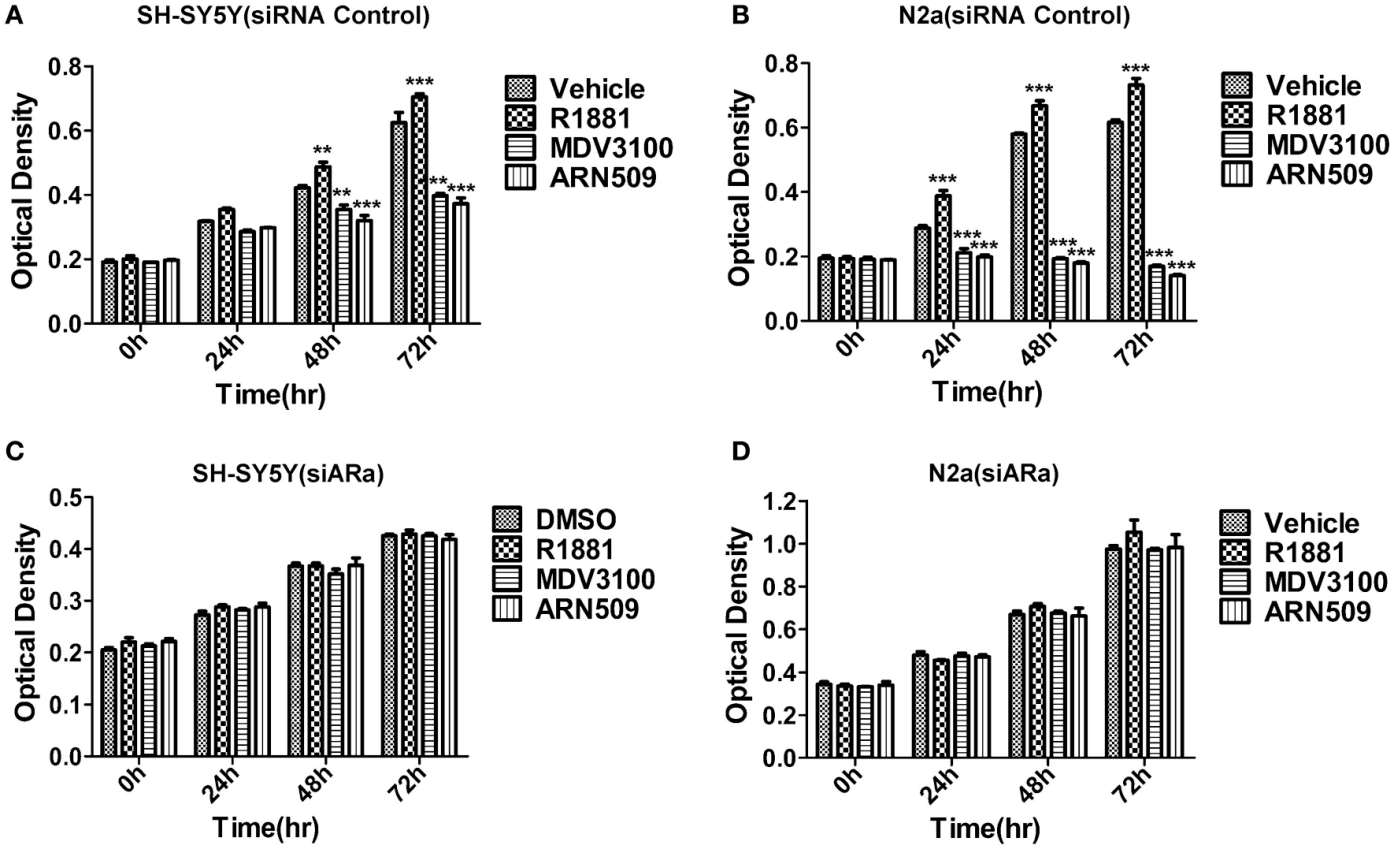
**The effects of androgen (R1881) and the antagonists of AR (ARN509 and MDV3100) on the proliferation of SH-SY5Y and N2a cells. (A,B)** After incubation of the two cell lines (SH-SY5Y and N2a) for 0, 24, 48, and 72 h with R1881 (10 nM) or MDV3100 (10 μM) or ARN509 (10 μM), cell proliferation was determined using MTT assay. Results are expressed as the optical density of the wells on a 96-well dish. **(C,D)** 72 h after siRNA transfection, the same treatment was performed as above. Data represent an average of three independent experiments. The data are reported as mean ± SD (^**^ *P* < 0.01, ^***^ *P* < 0.001).

We first studied the *in vitro* effect of AR signaling on the cell proliferation of SH-SY5Y and N2A cells lines via MTT assays. The neuroblastoma cells were treated with R1881 (10 nM), a synthetic agonist of AR, or the antagonists of AR, ARN509 (10 μm), and MDV3100 (10 μm) for 72 h. The results showed that R1881 promoted the proliferation of SH-SY5Y and N2a, while the antagonists of AR dramatically inhibit the proliferation of the two cell lines (Figures 2A,B). On the other hand, there was no significant difference of the proliferative activity when AR knockdown cells treated with AR agonist or antagonists in the two neuroblastoma cell lines (Figures 2C,D). Besides, inhibition of AR expression with siRNA led to decreased cell proliferation in both cell lines.

Vehicle (DMSO)-treated cells grew well, but slow, that suggests AR activity is important but not the unique factor to neuroblastoma cells' growth. Besides, it suggested neuroblastoma cells produce hormones to induce the growth of their own, which explained the nuclear expression of AR with vehicle treatment (Figure 1F) as well as administration of AR antagonists can achieve better growth inhibition compared with the vehicle (Figures 2A,B).

### R1881 promotes and AR antagonists inhibit the migratory ability of neuroblastoma cell lines

**Figure 3:**
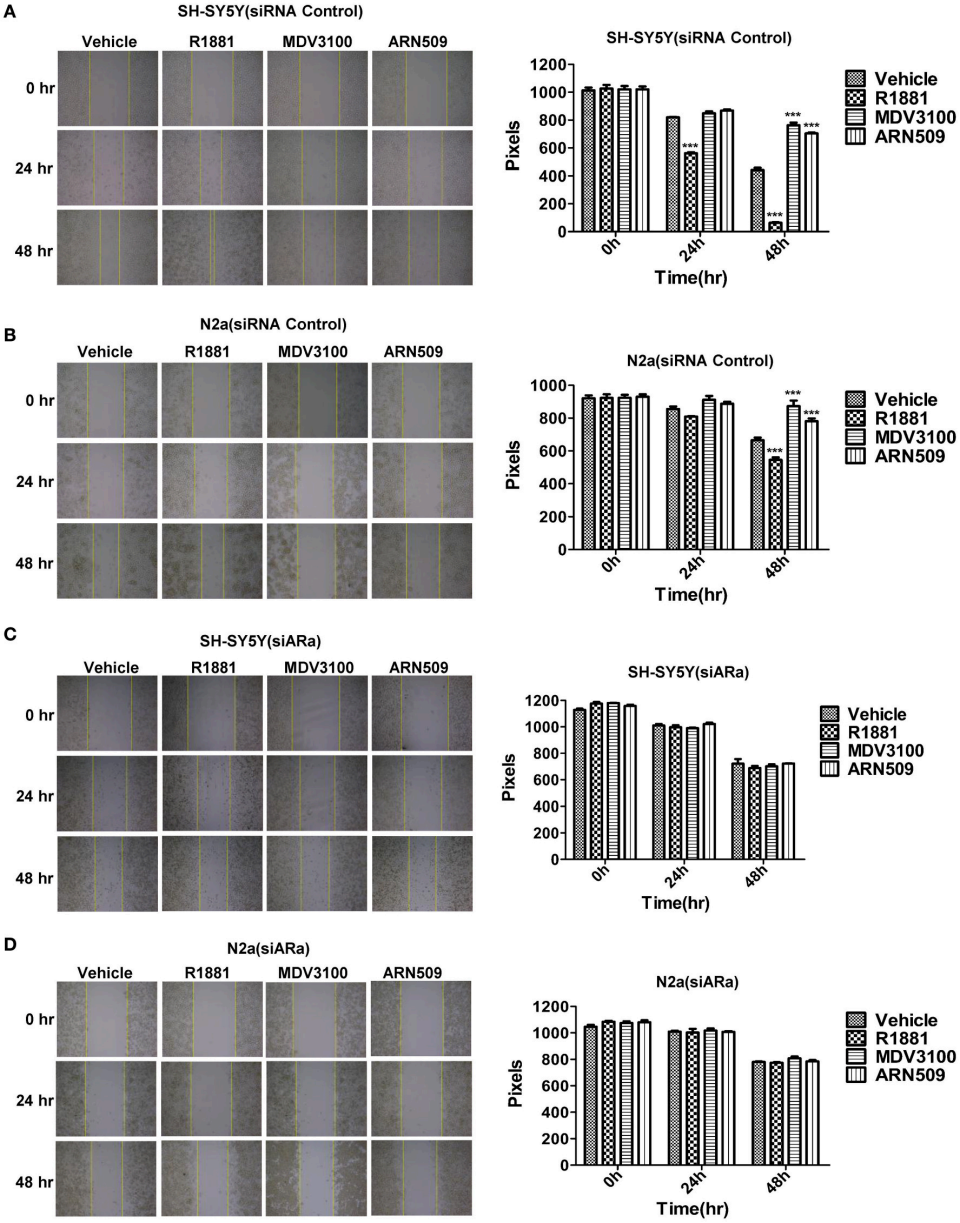
**The effects of androgen (R1881) and the antagonists of AR (ARN509 and MDV3100) on the migration ability of SH-SY5Y and N2a cells. (A,B)** A scratch was made in a six-well plate of confluent SH-SY5Y and N2a cells using a 200 μl tip, images were taken at *T* = 0, 24, and 48 h with indicated treatment, and quantification of the distance of the wound closure was assessed over time using Image-Pro Plus 6.0. **(C,D)** 72 h after siRNA transfection, the same treatment was performed as above. Results are expressed as the pixels between the two lines. Data represent an average of three independent experiments. The data are reported as mean ± SD (^***^P < 0.001).

A wound-healing assay was used to evaluate the effect of R1881 and the AR antagonists, ARN509, and MDV3100, on migration of both cell lines. R1881 obviously promoted cell migration and the cells were able to almost close the wound at 48 h, while both ARN509 and MDV3100 significantly inhibited the migration of the treated cells (Figures 3A,B). However, the migratory ability of neuroblastoma cells was largely reduced by siARa (Figure 3A vs. Figure 3C and Figure 3B vs. Figure 3D, in vehicle and R1881-treated groups). The result also demonstrated that AR knockdown attenuated the agonist effect of R1881 and the antagonist effect of MDV3100 and ARN509 (Figures 3C,D). These showed R1881 promote the migratory ability of both neuroblastoma cell lines while AR knockdown, ARN509 and MDV3100 suppress the migratory ability.

### Androgen promotes and AR antagonists inhibit the invasive ability of SH-SY5Y cells

**Figure 4:**
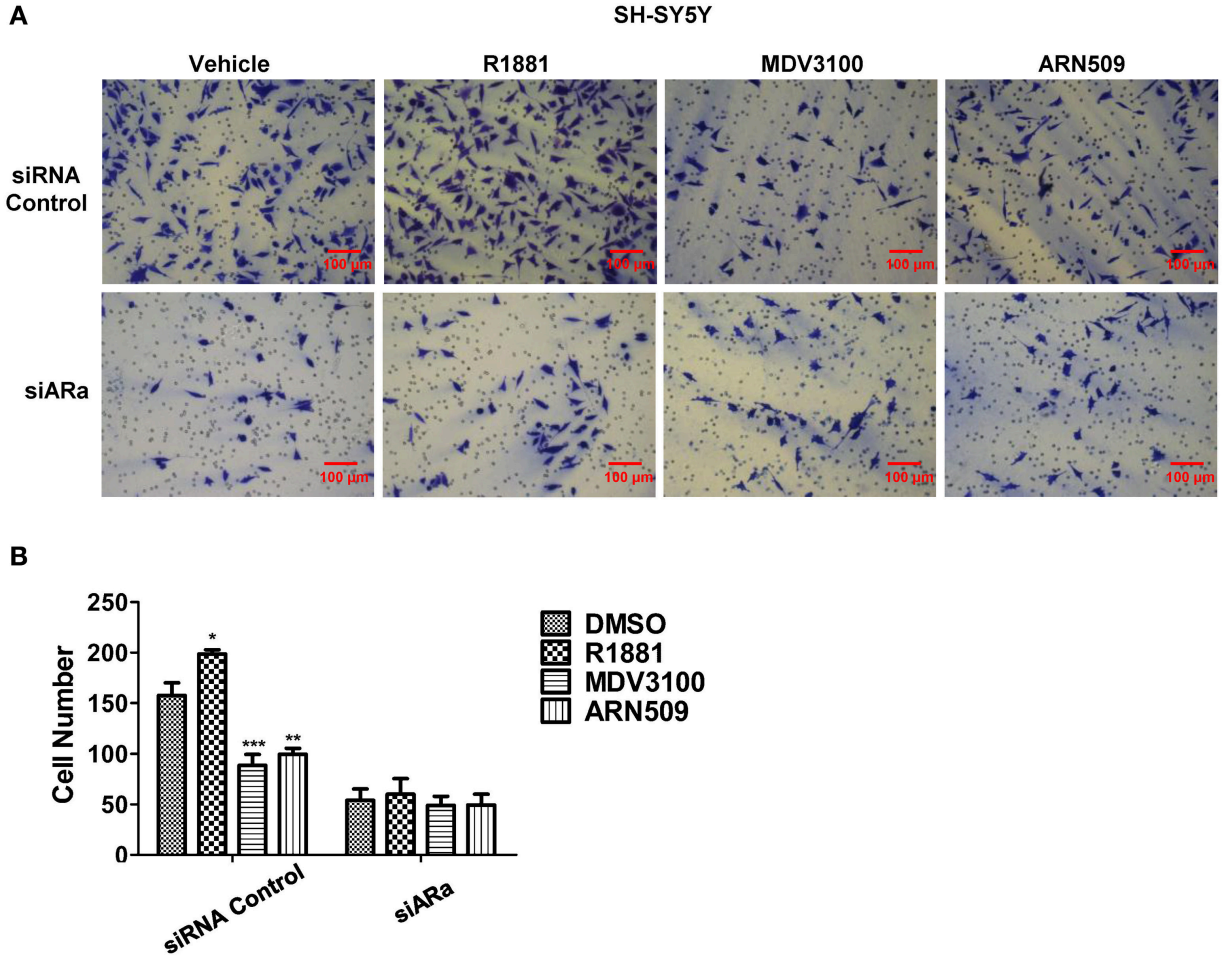
**The effects of androgen (R1881) and the antagonists of AR (ARN509 and MDV3100) on the invasion of SH-SY5Y cells**. **(A,B)** SH-SY5Y cells were seeded onto the Matrigel^*TM*^-coated membrane in the top chamber of the trans-well with indicated treatment in the presence of 5% cFBS in the lower chamber. Cells that invaded to the lower chamber after 48 h were fixed with paraformaldehyde, stained with 0.005% crystal violet, counted and represented as a number of invaded cells. Scale bar = 100 μm. 72 h after siRNA transfection, the same treatment was performed as above. Data represent an average of three independent experiments. The data are reported as mean ± SD (^*^P < 0.01, ^**^P < 0.01, ^***^P < 0.001).

Next, we use Matrigel™-coated trans-well experiments to detect cell invasion. R1881 obviously increased the amount of invasive cells compared with DMSO or AR antagonists treatment (Figures 4A upper panel, **B**). Interestingly, depletion of AR by siRNA attenuated the effect of either R1881 or AR antagonist (Figures 4A lower panel, **B**). These results indicated that the invasive abilities of SH-SY5Y cell line were closely related to AR signaling activation. The assay was not performed on N2a cells since they failed to show an ability to invade when using FBS as a chemo-attractant.

### Androgen promotes cell sphere formation of neuroblastoma cells while antagonist of androgen receptor inhibits cell sphere formation

**Figure 5:**
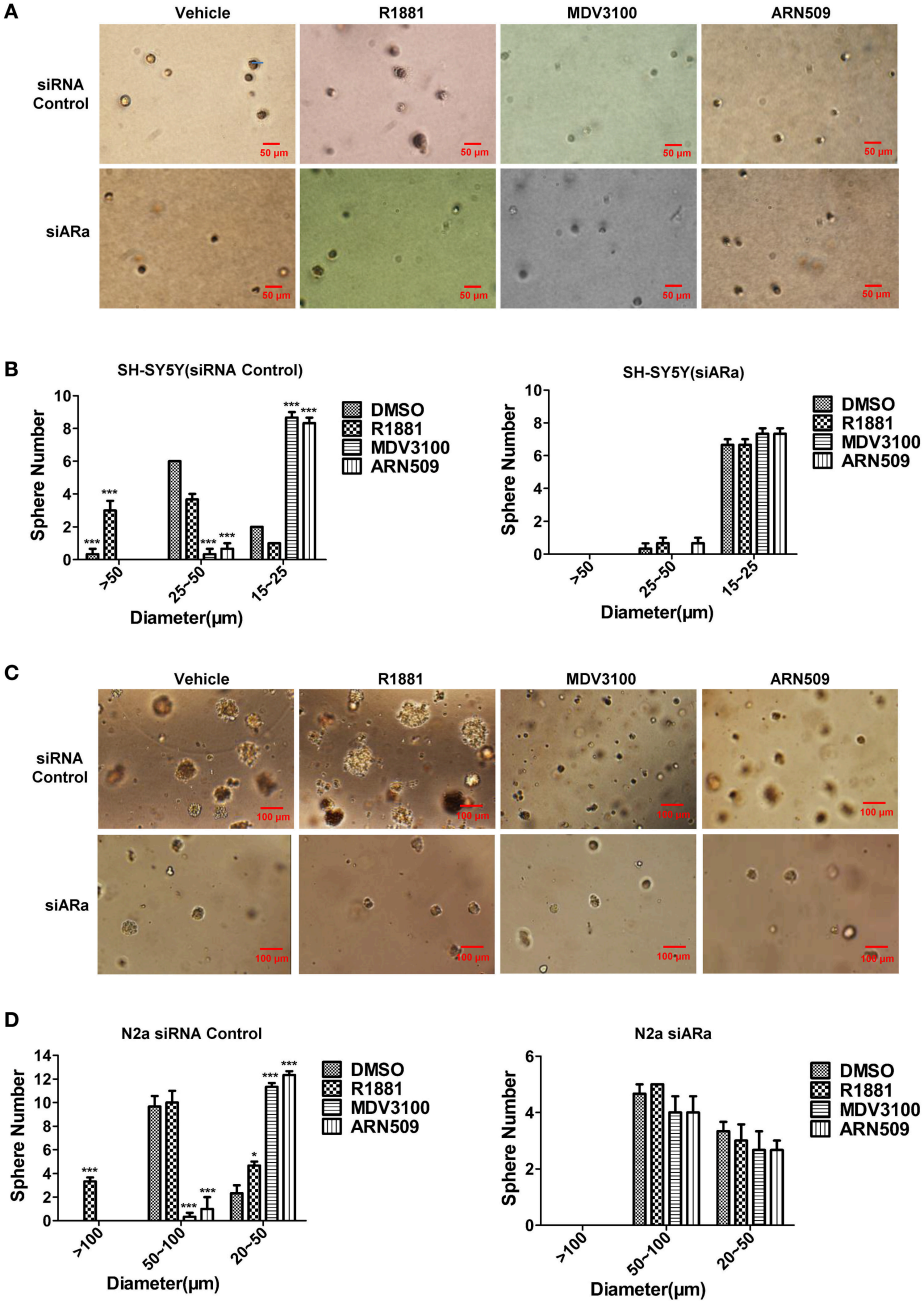
**The effects of androgen (R1881) and the antagonists of AR (ARN509 and MDV3100) on the sphere-forming ability of SH-SY5Y and N2a cells. (A,B)** Representative bright-field images of SH-SY5Y spheres with indicated treatment. Images were visualized by inverted microscope at 10x magnification. Scale bar = 50 μm. According to the size, the spheres are divided into three groups and their diameters were auto-calculated by Image-Pro Plus 6.0. Data represent an average of three independent experiments. **(C,D)** N2a spheres, 72h after siRNA transfection, the same treatment was performed as above. Scale bar = 100 μm. The data are reported as mean ± SD (^*^P < 0.05, ^**^P < 0.01, ^***^P < 0.001).

In order to better visualize the sphere-forming capabilities of neuroblastoma cells, single cell suspensions of SH-SY5Y and N2a were cultured in soft agar and Matrigel™ for 21 and 7 days, respectively. The spheres were then visualized under an inverted light microscope and bright field images were acquired. Both cell lines treated with R1881 produced more and relatively larger spheres, while cells treated with AR antagonists produced significantly smaller spheres, compared to those cells treated with vehicle (Figures 5A,C, upper panles). Inhibition of AR expression by siRNA obviously blocked the promoting or suppressing effect of R1881 or AR antagonists, respectively on sphere formation of both cell lines (Figures 5B,D).

### Androgen promotes neuroblastoma xenograft tumor growth

**Figure 6:**
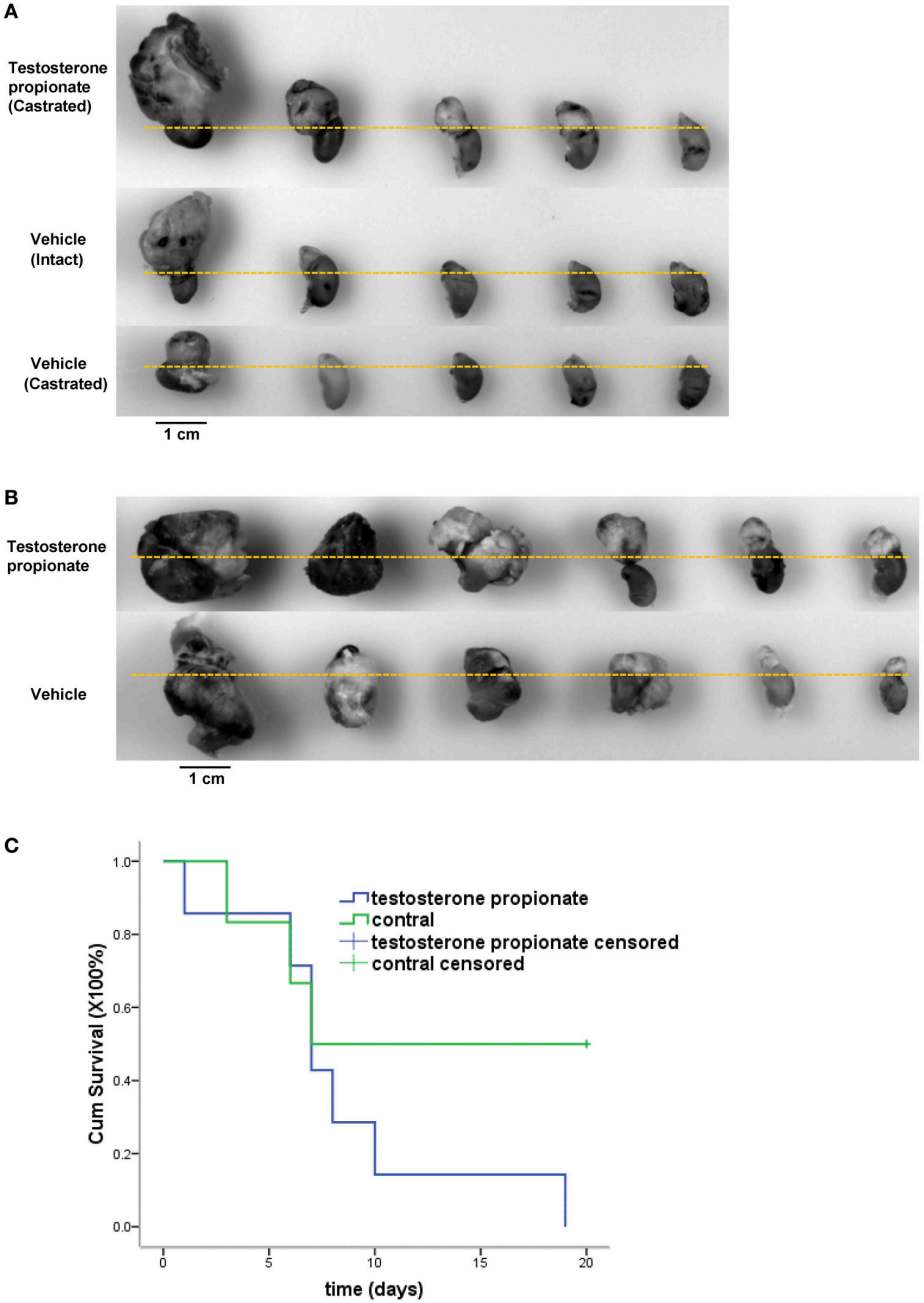
**Testosterone propionate promoted N2a tumor growth *in vivo***. Mice bearing adrenal N2a xenograft tumors were treated with testosterone propionate or vehicle and the tumors were harvested together with the kidneys at the end of the experiment. **(A,B)** Representative pictures of harvested tumors are shown in groups. The kidneys were placed below the dashed lines. **(C)** Cumulative survival among N2a bearing castrated male mice treated with testosterone propionate (*n* = 6) or vehicle (*n* = 7).

To further evaluate the efficacy of androgen on cancer cells growth *in vivo*, N2a neuroblastoma cells were injected into the adrenal glands of nude mice, and mice were treated with either vehicle alone or testosterone propionate dissolved in the vehicle. Testosterone propionate treatment resulted in increased tumor volume in both male and female mice (Figures 6A,B) as well as shorter survival time in male mice (Figure 6C). These results indicated the proliferative effect of androgen in N2a xenograft models.

## Discussion

The present study indicated that AR agonist R1881 obviously promoted cell proliferation, migration, invasion and sphere formation of both neuroblastoma cell lines, SH-SY5Y and N2a, *in vitro* and the progression of N2a *in vivo*. While the antagonists of AR, MDV3100 and ARN509, played an opposite role *in vitro*. Moreover, the promoting and suppressing effects of AR agonist and antagonists, respectively, had been evidently attenuated by inhibition of endogenous AR expression, suggesting that AR signaling pathway may take part in the progression of neuroblastoma. However, the underlying mechanism is still uncertain and will be illustrated in our future studies. The two AR antagonists in the treatment of prostate cancer have achieved good clinical curative efficacy, therefore they are expected to become the new drugs for the treatment of neuroblastoma in the preclinical study.

Androgen and androgen receptor (AR) signaling has an important role in the initiation and progression of many hormone-related cancers including prostate and breast cancer (Chang et al., 2014). AR is primarily responsible for mediating the physiological effects of androgens by binding to specific DNA sequences, androgen responsive element (ARE), which is present in regulatory elements on the AR-responsive target genes, such as vascular endothelial growth factor (VEGF) (Bolton et al., 2007), matrix metalloproteinases (MMPs) (Joshi et al., 2007), and tubulin (Mariani et al., 2015).

First of all, it's well accepted that cell proliferation and migration are mainly regulated by receptor tyrosine kinase ligands such as, a major contributor to angiogenesis, VEGF (Roy Choudhury et al., 2012). Previous studies have shown that VEGF and its receptors are expressed in human neuroblastoma tumors and cell lines, and several experimental therapeutic strategies have been emerged to target the interaction of VEGF with its receptors and thereby to suppress the growth of neuroblastoma (Ribatti and Ponzoni, 2005). More than that, the AR binding sites were discovered in the VEGF promoter and *in vivo* binding of AR to these sites was demonstrated by chromatin immunoprecipitation (Eisermann et al., 2013). Concerning that VEGF, an angiogenesis stimulator, has been detected in neuroblastoma tumors (Amoroso et al., 2015), we inspected the necrosis areas on N2a xenograft tumor (shown in Figure 6A) sections which may be consequences of inadequate blood supply. Unsurprisingly, testosterone propionate treated tumors exhibited less necrosis areas compared with vehicle treated tumors (Supplemental Figure 1A) in both male and female mice groups (Supplemental Figure 1B), suggesting an angiogenesis potential of AR signaling. Therefore, we speculate that activation of AR signaling induces VEGF production so as to promote proliferation and migration of the neuroblastoma cells.

On the other hand, it has been reported that the naturally occurring endogenous angiogenesis inhibitors affect neuroblastoma growth *in vivo*, especially the tissue inhibitors of matrix metalloproteinases (MMPs) (Joshi et al., 2007). In addition, MMPs express in human neuroblastoma and in advanced stages of neuroblastoma, and tumor cells secret MMPs favoring degradation of extracellular matrix and enhancing tumor dissemination (Roy Choudhury et al., 2012). Thus, MMPs facilitate invasion and migration of cancer cells. Furthermore, it has been confirmed that androgen response elements (AREs) involved in androgen-induced MMP-2 expression and MMP-2 expression is blocked by the androgen antagonist bicalutamide at the mRNA level (Li et al., 2007). Besides, MMP-2, MMP-9 and MMP-7 expression were confirmed in the SH-SY5Y cells (Lu et al., 2009). MMP9 is also a downstream target gene of AR (Li et al., 2015). So, in the present study, MMPs may contribute to the invasion ability of SH-SY5Y. Nevertheless, expression of endogenous MMP-2, MMP-9, and TIMP-1 (tissue inhibitor of matrix metalloproteinase 1) was not detected or at times barely detectable in the non-differentiated and differentiated N2a cells (Sbai et al., 2008). All above explains the difference of the invasive ability between SH-SY5Y and N2a. Thus, we propose another mechanism that AR signaling increases MMPs expression and consequently promotes invasion of neuroblastoma cells.

Furthermore, a major protein up-regulated in response to androgens in regenerating motor neurones is tubulin, the structural component of microtubules (Matsumoto et al., 1993). Previous experiments have confirmed that testosterone treatment of proliferating SH-SY5Y cells resulted in an increase in the level of the two tubulin subunits alpha- and beta-tubulin, and anti-androgens counteract the effect of testosterone on tubulin (Butler et al., 2001). Moreover, Tubb3, a beta-tubulin isotype, was found a direct target of AR and the AREs was determined (De Gendt et al., 2011) Beyond that, the protein interaction of AR with tubulin was reported facilitating nuclear translocation of AR (Zhu et al., 2010). Thus, we can reasonably infer activated AR signaling may induce tubulin expression leading to neuroblastoma growth.

In conclusion, we have demonstrated that the androgen effectively promotes the progression of neuroblastoma tumor cells. More important, our result indicates the anti-tumor role of MDV3100 and ARN509 on neuroblastoma cells by targeting AR as well as provides insight into the anti-androgen treatment of neuroblastoma.

## Author contributions

Conceived and designed the experiments: YW, RX, SF; Performed the experiments: JS, DW, LG; Analyzed the data: YW, JS, and RX; Manuscript writing: YW, JS, SF.

## Funding

The study was funded by the National Natural Science Foundation of China (NSFC 31201070) and China Postdoctoral Science Foundation (204001-5) to Yang Wang.

### Conflict of interest statement

The authors declare that the research was conducted in the absence of any commercial or financial relationships that could be construed as a potential conflict of interest. The reviewer JH and handling Editor declared their shared affiliation, and the handling Editor states that the process nevertheless met the standards of a fair and objective review.

## Supplementary material

Supplementary Figure 1 **Testosterone propionate decreased the necrosis of N2a tumor *in vivo*. (A)** Sections of harvested N2a xenograft tumors from mice treated with vehicle and testosterone propionate were formalin-fixed-paraffin-embedded and stained with hematoxylin and eosin (HE). N, necrosis area. Scale bar = 100 μm **(B)** The areas occupied by necrosis on indicated sections were statistically calculated using Image Pro-Plus 6.0. 5 pictures of views were taken randomly on an HE stained section, tumor sections from 3 mice were analyzed in each group. T.P., testosterone propionate. The data are reported as mean ± SD (^***^*P* < 0.001).
