# Supplementary material for: Androgen Receptor Regulates the Growth of Neuroblastoma Cells in vitro and in vivo
Source: Front Neurosci. 2017 Mar 7;11:116. doi: 10.3389/fnins.2017.00116 (PMC5339338; doi:10.3389/fnins.2017.00116)
Supplement: Supplementary Figure 1 — Testosterone propionate decreased the necrosis of N2a tumor in vivo. (A) Sections of harvested N2a xenograft tumors from mice treated with vehicle and testosterone propionate were formalin-fixed-paraffin-embedded and stained with hematoxylin and eosin (HE). N, necrosis area. Scale bar = 100 μm (B) The areas occupied by necrosis on indicated sections were statistically calculated using Image Pro-Plus 6.0. 5 pictures of views were taken randomly on an HE stained section, tumor sections from 3 mice were analyzed in each group. T.P., testosterone propionate. The data are reported as mean ± SD (***P < 0.001). [file Image1.PDF]

A

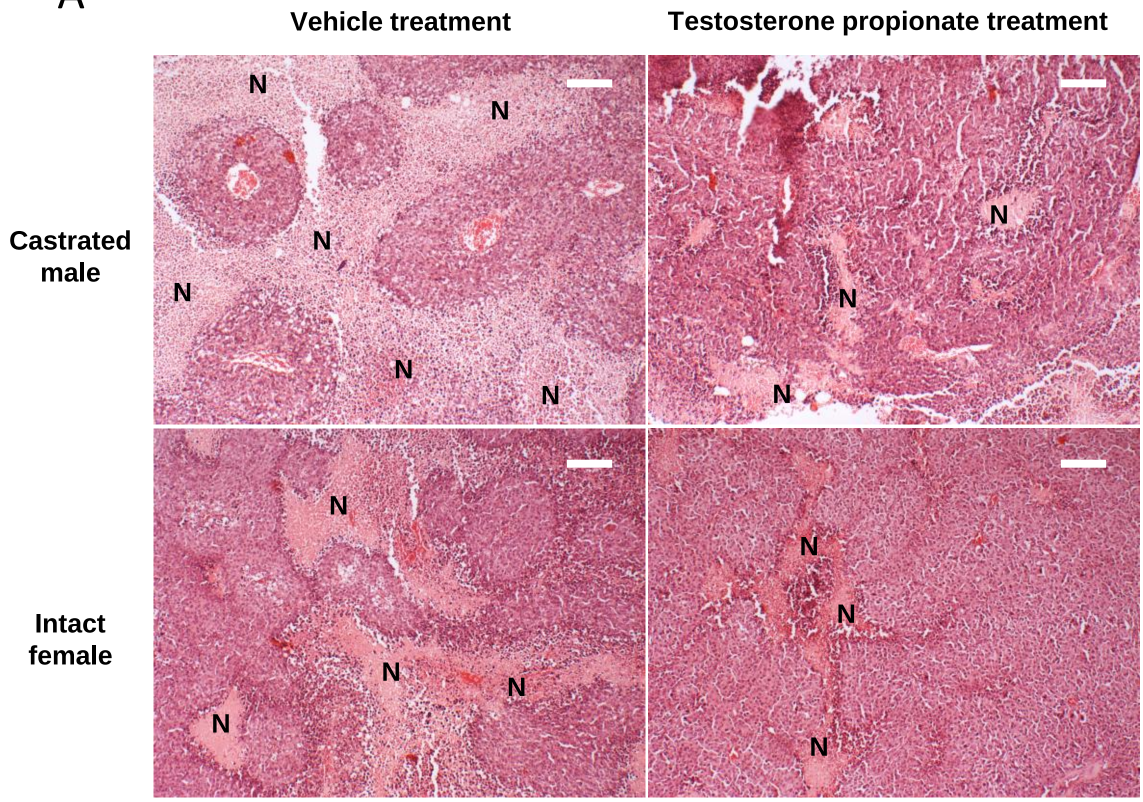

B

	Necrosis area (%) ± SD	
	**Vehicle**	**T.P.** ***
**Castrated male**	48.6±12.1 (n=3)	13±11.6 (n=3)
**Intact female**	41.5±9.3 (n=3)	17±8.9 (n=3)

T.P., testosterone propionate. The data are reported as mean ± SD (***P<0.001).
